# Costs and barriers faced by households seeking malaria treatment in the Upper River Region, The Gambia

**DOI:** 10.1186/s12936-021-03898-6

**Published:** 2021-09-16

**Authors:** Henk Broekhuizen, Alexandra Fehr, Claudia Nieto-Sanchez, Joan Muela, Koen Peeters-Grietens, Tom Smekens, Momodou Kalleh, Esmé Rijndertse, Jane Achan, Umberto D’Alessandro

**Affiliations:** 1 Dept. Health Evidence, Radboud University Medical Center, Nijmegen, The Netherlands; 2 Dept. Health and Society, Wageningen University and Research, Wageningen, The Netherlands; 3 Department of Sociology and Anthropology, Faculty of Social and Behavioural Science, University of Amsterdam, Amsterdam, The Netherlands; 4 Medical Anthropology Unit, Department of Public Health, Institute of Tropical Medicine, Antwerp, Belgium; 5 PASS Suisse, Neuchâtel, Switzerland; 6 School of Tropical Medicine and Global Health, Nagasaki University, Nagasaki, Japan; 7 Department of Public Health, Institute of Tropical Medicine, Antwerp, Belgium; 8 National Malaria Control Programme, Banjul, The Gambia; 9 MRC The Gambia at the London School of Hygiene & Tropical Medicine, Fajara, The Gambia; 10 Malaria Research Consortium, London, UK

## Abstract

**Background:**

Malaria transmission in The Gambia decreased substantially over the last 20 years thanks to the scale-up of control interventions. However, malaria prevalence is still relatively high in eastern Gambia and represents both a health and a financial burden for households. This study aims to quantify the out-of-pocket costs and productivity losses of seeking malaria treatment at household level.

**Methods:**

A household survey was carried out through in-person interviews. Respondents were asked about malaria prevention methods, their treatment-seeking behaviour, and any costs incurred for transport, services, food, and/or overnight stays. A bottom-up costing approach was used to calculate the unit cost of treatment and a tobit regression approach to investigate cost drivers.

**Results:**

The survey included 864 respondents, mainly subsistence farmers. Most respondents (87%) considered malaria to be a problem affecting their ability to perform their regular duties. Respondents preferred going to a health facility for treatment. The primary reason for not going was related to costs; 70% of respondents incurred costs for seeking health care, with a median of £3.62 (IQR: £1.73 to £6.10). The primary driver of cost was living in one of the villages that are off the main road and/or far from health facilities. 66% reported productivity loss of 5 working days on average during a malaria episode of them or their child.

**Conclusions:**

Although malaria prevalence is decreasing and treatment is provided free of charge, households seeking treatment are confronted with out-of-pocket expenditures and lost working days; particularly in remote villages.

## Background

In 2017, the estimated global burden of malaria was 219 million cases and 435,000 deaths [1], 90% of which occurring in sub-Saharan Africa [2]. The Gambia, a small country in western Africa, has been very successful in decreasing the malaria burden: clinical malaria incidence has dropped fivefold, from 275 cases per 1000 population in 2010 to 57 cases per 1000 population in 2017. This has been achieved thanks to the scale-up by the National Malaria Control Programme (NMCP) of malaria control interventions, such as insecticide-treated nets, indoor residual spraying (IRS), intermittent preventative treatment during pregnancy, and seasonal malaria chemoprevention (SMC). Nevertheless, malaria transmission is still ongoing, particularly in eastern Gambia, in the Upper River Region (URR) [3]. Asymptomatic malaria cases are often mentioned as the reservoirs for this continued transmission and novel strategies are needed to clear these [2–4].

Several trials are currently underway to investigate if such residual transmission can be reduced with mass drug administration (MDA) based on the endectocidal drug ivermectin (IVM) [4–6]. To evaluate adoption of such an approach by national or global stakeholders, it is important that its cost-effectiveness is established. Costing studies are an essential component of cost-effectiveness analyses [7, 8]. Although various types of costing studies are needed, household costs are a particularly important type of costing study because apart from a health impact, malaria also has a financial impact on households because they incur costs for prevention and for case management. Malaria diagnosis and treatment in The Gambia, as in many other sub-Saharan African countries, is provided free of charge, but indirect costs can still be incurred. Studies from Ghana, Kenya and Ethiopia show that households incur costs for transport, food, and overnight stays in health facilities [9–11]. Unfortunately, household costing data are not available for The Gambia. The aim of this study is to fill this knowledge gap by surveying households in URR about their malaria treatment-seeking behaviour and the (in)direct costs they incur whilst doing so.

## Methods

### Study setting and respondent selection

This study was part of a larger village-level, cluster-randomized trial evaluating the efficacy of MDA with IVM and the anti-malarial drug dihydroartemisinin-piperaquine (DP) on residual malaria transmission (#NCT03576313). The trial started in September–October 2018 and included 32 medium-sized villages (16 control and 16 intervention) in the southern bank part of the URR. As part of the RCT, a quantitative survey on the acceptability of MDA and the (costs of) seeking care was carried out. The sample size was designed to substantiate inferences concerning trial participation, defined as a multinomial outcome with three levels: no participation, partial participation, full participation. Assuming an effect size of 1.5, an intra-correlation of 1 and 80% power, the required sample size was estimated at 850 individuals (aged above 12), rounded up to 900, in the 16 intervention villages. The target sample size per village was made proportional to the village population size. Participants were randomly selected from the most recent census data collected by the trial field team; individuals absent or refusing to participate were replaced by others of similar age and gender. In addition to the household survey, village health workers (VHWs) of trial intervention villages were interviewed. The aim was to include all 16 intervention villages.

### Data collection

The survey was carried out between January and February of 2019, 2–3 months after the first intervention year. Part of the acceptability survey concerned treatment-seeking behaviour and costs. These questions were developed based on the ACT Consortium guidance [12]. Respondents, besides demographic information, general malaria knowledge, owned means of transport, and primary economic activities; were asked questions on their treatment-seeking behaviour for malaria, malaria prevention methods employed, and perceived problems associated with malaria. Information on the last episode of malaria experienced by them and by one child of the household was also collected, including the preferred and chosen treatment option and costs incurred. Questions and potential responses of non-economic topics were formulated on the basis of qualitative and ethnographic data collected prior to and during the implementation of the MDA in 2018. Data were electronically captured in Epi Info v.7 by trained field workers using Android tablets. Data were synced at the end of every field day and checked for quality.

The interviews with VHWs of trial intervention villages were conducted during the summer of 2019. They were asked questions about their background as well as their role in malaria testing and treatment. In addition, they were asked about barriers surrounding malaria testing and treatment facing them as well as the inhabitants of their village.

### Analysis of costing data

A bottom-up costing approach was used to calculate the unit costs incurred by a household of the last malaria episode for an adult and for a child. Direct cost components were defined as costs for testing and treatment while indirect costs were defined as costs of transport, costs of overnight stays and related food. Uncomplicated cases were assumed to not incur costs for overnight stays or food. All costs were obtained directly from the respondents. Productivity losses were measured in number of working days missed, either to obtain one’s own treatment or to accompany a sick child to a health facility. The total financial impact of malaria care-seeking on households in URR was calculated by multiplying the unit cost of seeking care by the average yearly clinical incidence of malaria based on data obtained from the NMCP.

Costs are reported in 1 January 2020 British pounds, using the average Gambian Dalasi to British Pound exchange rate during the period October–November 2018 (about 61 Dalasi per 1 GBP). This is the peak malaria season when malaria-related costs would have been incurred.

To investigate what demographic variables drive the household unit cost of obtaining malaria treatment, a tobit regression approach was applied [13]. This approach is particularly well-suited for costing data where a proportion of the sample may have zero costs, leading to highly skewed data.

### Ethics

All participants (and their guardians if under 18) were explained the purpose of the study by trained field staff in their preferred language prior to giving informed consent and/or assent (minors) to participate. The study was approved by the Institutional Review Boards of the Institute of Tropical Medicine Antwerp and by the Gambian Government/MRC Joint Ethics Committee.

## Results

The household survey was completed by 864 respondents, 66% of them women. The median age was 29 years (IQR: 19–41). Fulas were the most represented ethnic group (73%) and most respondents (80%) were subsistence farmers. A large proportion of respondents (41%) had not received any formal education (Table 1). In total, 14 VHWs were interviewed. Most had received a 4-week training on testing and treating Malaria in Basse (the major town in URR) and they had 5 years’ worth of experience on average.

**Table.1:** Demographics of the respondent population and results from the multivariate tobit regression against total costs in 2020 GBP

Descriptive statistics		Multivariate tobit regression^†^		
N = 864	Median (IQR)	Coefficient	Standard error	p-value
Age	29 (19–41)	− 0.33	0.78	0.67

	n (%)			
Gender = female	566 (65.8)	− 42.55	29.07	0.143
Village				
Sare Njobo	86 (10.0)	− 33.54	41.99	0.424
Sare Banico Gimara	29 (3.4)	63.78	54.42	0.241
Sare Cherno	41 (4.8)	− 118.19	54.29	0.029*
Jalali Kunda	36 (4.2)	12.53	57.31	0.827
Sare Jallow	40 (4.6)	− 88.37	50.77	0.082
Giroba	112 (13.0)	Reference level		
Sare Yoro Checky	36 (4.2)	− 34.61	53.15	0.515
Keneba	60 (7.0)	17.45	44.62	0.696
Sare Garba	38 (4.4)	184.35	49.31	0.000***
Koli Kunda	75 (8.7)	122.43	40.85	0.003**
Ceesay Kunda	54 (6.3)	79.82	43.88	0.069
Karantaba	38 (4.4)	66.91	47.19	0.156
Darsilameh Julah	35 (4.1)	175.12	54.09	0.001***
Sare Gela	70 (8.1)	92.36	42.77	0.031*
Sami Kuta	70 (8.1)	2.38	44.95	0.958
Sare Biru	43 (5.0)	16.82	49.43	0.734
Status				
Compound head	69 (8.0)	Reference level		
Household head	24 (2.8)	− 8.54	68.70	0.901
Compound member	226 (26.2)	− 56.73	39.24	0.148
Wife	360 (41.7)	− 4.54	44.99	0.920
Child	182 (21.1)	− 69.01	49.36	0.162
Other	2 (0.2)	− 122.17	217.87	0.575
Education				
None	357 (41.1)	Reference level		
Standard^‡^	243 (28.2)	− 23.34	25.07	0.352
Quranic	261 (30.2)	− 63.13	21.95	0.004**
Other	2 (0.2)	− 154.83	155.49	0.319
Ethnic group		Not included in regression		
Fula	626 (72.5)			
Mandinka	172 (19.9)			
Sarahule	56 (6.5)			
Other	9 (1.0)			
Primary economic activity				
Farming	694 (80.3)	− 3.07	29.65	0.918
Herding	65 (7.5)	− 7.99	36.19	0.825
Business	131 (15.2)	70.76	25.81	0.006**
Domestic	263 (30.0)	− 10.21	20.26	0.614

*IQR* interquartile range

^†^Intercepts of model had as estimates 262.64 and 5.30 with standard errors of 68.05 and 0.03; both had p-values < 0.01

^‡^Standard education was defined as having enjoyed primary, junior, senior, and/or more than senior education

Asterisks denote significance level: *p ≤ 0.05, **p ≤ 0.01, ***p ≤ 0.001

### Perspectives on malaria and healthcare-seeking behaviour

A large proportion of respondents (63%) considered malaria a relevant, but declining problem, while 25% had the opposite view and considered malaria as a current problem. This view was shared by VHWs, of whom 79% indicated malaria was a problem in their village. When asked what non-health problems malaria can cause for the patient or their family, 65% stated missing work, 56% missing household responsibilities, and 50% missing school. Malaria was indicated to cause financial problems due to costs incurred at the health facility (42%), for medicines (46%), or for transport (37%).

The belief that malaria can be prevented by using bed nets was shared by 95% of participants. In addition, almost half of respondents (45%) believed malaria could be prevented by cleaning the environment. Fewer believed malaria could be prevented using sprays (20%), coils (11%), medication (6%), or herbs (13%). Regarding treatment, 85% of respondents believed malaria medication should be taken even if someone was no longer visibly sick. VHWs indicated that the majority of people in their villages used bed nets (33%), environmental cleaning (21%), or coils (16%) to prevent malaria. All VHWs indicated that NMCP prevention activities (bed net campaign, IRS, SMC) had taken placed in their village in 2019. Most VHWs had a role in IRS and SMC (> 80%), but this was less so for net distribution (57%).

**Fig. 1:**
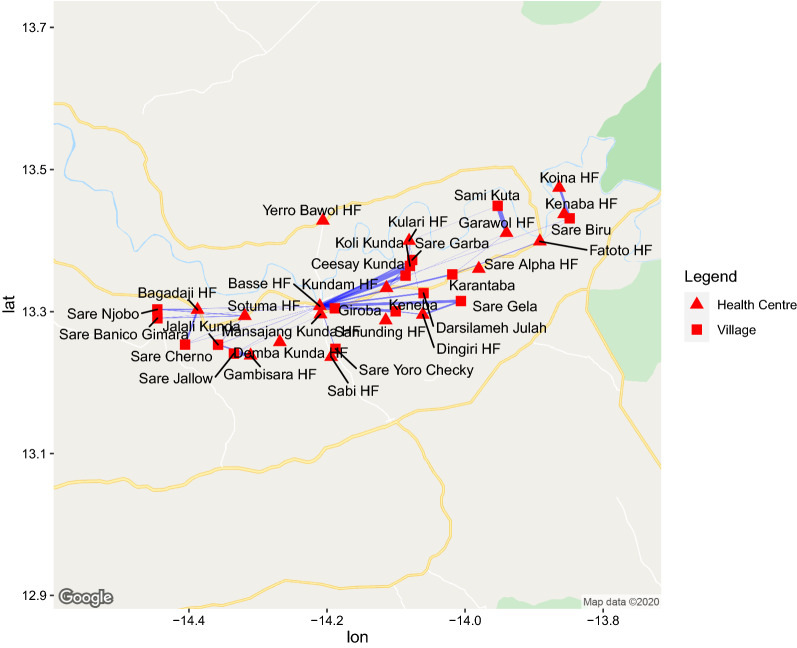
Map of the Upper River Region in the Gambia showing the health facilities visited for malaria treatment by survey respondents. The width of lines is proportional to the number of respondents from a particular that indicated having gone to a particular health facility

Malaria in the previous months was reported by 28% of adults; 37% of them reported an episode of malaria among children of their household. All but one VHWs indicated that most villagers with fever came to them first to seek testing and/or treatment. Despite this, the large majority of episodes in adults (89%) and children (91%) reported preferring and having received treatment at a health facility. Very few adults self-treated (4%), received treatment by VHWs (4%), or by a traditional healer (1%). Similarly, most children (91%) were treated at a health facility. Overall, most respondents went to the centrally located—and largest—facility in Basse, despite not being the closest one for many respondents (Fig. 1).

Most respondents travelled to the health facility using public transport (36%), followed by motorcycle (24%), walking (12%), or donkey cart (8%). Private or MRC-provided transport were rarely used (1% and < 1%, respectively). 15% of respondents used a combination of transport modes. The choice of transport was not different between adult cases versus paediatric cases. The mean distance to the chosen health facility was 37 km (95% CI 12 to 48).

### Barriers to preventing malaria and seeking care

Not getting/having enough bed nets (50%) and financial difficulties (29%) were mentioned by VHWs as most important prevention challenges, followed by a lack of cleaning materials (21%) or coils (14%). The 59 respondents who indicated that they did not go to the health facility for the most recent malaria episode gave as reasons: transport costs (17%), service costs (12%), or that they were treated at home (10%). All VHWs indicated that a lack of supplies prevented them from consistently testing and treating malaria as per their training and the village’s needs. Barriers to accessing care for villagers according to VHWs were the unavailability of RDTs (86%) and anti-malarial drugs (93%) at the village level. Although a majority (86%) of VHWs indicated that the coordination between them and the health facilities was good, 64% of them also stated that having to travel to the health facility presented a barrier to accessing malaria care for people in their village.

### Cost of seeking treatment at a health facility

More than half (54%) of respondents who attended a health facility incurred out-of-pocket expenditures for transport, with a median cost of £1.07 (IQR: £0.66 to £1.65). When at the health facility, 65% had to pay for services, to the median amount of £3.30 (IQR: £1.65 to £4.94). Only 6% of respondents had to pay for food during the visit to the health facility; the median amount paid was £4.37 (IQR: £1.65 to £5.15). When combined, 70% of respondents incurred out-of-pocket expenses when attending a health facility, the median of which was £3.62 (IQR: £1.73 to £6.10). Regarding productivity losses, 56% of respondents indicated productive work lost during their last malarial episode and 39% indicated productive work lost during the last episode of their child. In both cases, 5 working days were lost on average by the adult (IQR: 4 to 7).

The total expected expenditures by households in URR on malaria treatment ranged from £44,293 in 2017 to £164,288 in 2015 (Table 2). Of these costs, 97% would be for uncomplicated cases. Total expected expenditures were calculated by multiplying the out-of-pocket cost estimates for seeking care with the historical clinical incidence (of both uncomplicated and complicated malaria), as reported by NMCP.

**Table.2:** Estimation of total costs incurred by households in URR during the years 2013–2017 based on this study and malaria clinical incidence data in URR for these years obtained from the Gambian National Malaria Control Programme

Years	Complicated cases	Uncomplicated cases	Household costs for seeking treatment
2013	1515	45,479	£145,021
2014	933	26,806	£85,613
2015	1273	52,007	£164,288
2016	1262	34,167	£109,368
2017	526	13,821	£44,293

### Drivers of costs

Overall, services constituted the majority of costs (69%) followed by transport (22%) and overnight stays/food (9%). Median costs were higher for adults (£4.20, IQR: £2.02 to £6.70) than for children (£3.54, IQR: £0.41 to £6.18). The average out-of-pocket cost incurred for services differed by the choice of the health provider; it was the highest for a health facility (£3.91), followed by home treatment (£3.21), village health worker (£2.73), traditional healer (£2.06) or MRC (£2.04). Travelling by public transport to attend a health facility costed an average amount of £1.34 out-of-pocket. This was slightly less for those who travelled by motorbike (£1.21), donkey cart (£0.33), or walking (£0.03). Transport expenditures for respondents who used multiple modes of transport were £1.30 on average.

**Fig. 2:**
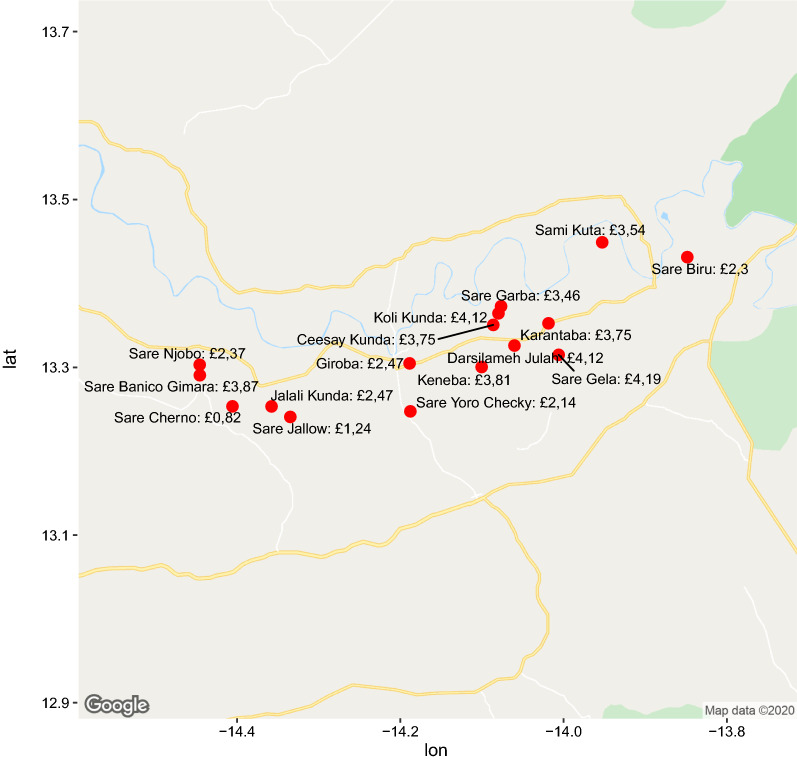
Map of the Upper River Region showing median household costs for seeking malaria care during the last transmission season per village as indicated by survey respondents

For the tobit regression analysis, the demographic variables in Table 1 were included, with the exception of ethnic group as this is highly correlated with village of residence (most villages are composed predominantly of one ethnic group). The cost of seeking malaria care is reduced significantly by having a Quranic as opposed to no education (p < 0.01) and having business among one’s primary economic activities (p < 0.01). Compared to the largest village of Giroba (Fig. 2), people from some villages (Sare Garba, Koli Kunda, Darsilameh Julah or Sare Gela) bear a significantly higher total cost (p < 0.01), while villagers from Sare Cherno had a significantly lower cost (p = 0.03). Age, status in household, and owning particular modes of transport did not significantly impact the total costs of seeking malaria treatment.

## Discussion

This study investigated treatment-seeking behaviour and expenditures incurred by households in URR, The Gambia, when one of their members contracts malaria. The results show that most respondents considered malaria a problem that affects their quality of life and their economic productivity. Although in The Gambia malaria treatment is free of charge, many barriers to this actually happening are mentioned. Seventy per cent of respondents reported incurring expenditures for malaria treatment. The health facility is seen as the most efficient way to treat malaria and households incurred a median of £3.72 in out-of-pocket expenses to obtain treatment there, two thirds of which was for services.

The primary cost driver in this study is village of residence. This is not surprising given the fact that the villages where respondents incurred significantly more costs are located between the health facilities surrounding Basse and the health facilities at the eastern tip of URR (Fig. 2). Furthermore, they are off the main west-east road and/or have a road connection that is in bad condition (especially during the rainy season). Village of residence was, in this study, a pragmatic surrogate for travel time because travel times are hard to calculate given the many modes of transport people employ and the varying condition of roads throughout the year. That travel time (or distance to health facility) is an important driver of costs is also found in earlier malaria treatment costing studies in Ghana, Ethiopia, Malawi, and Uganda [9, 11, 14, 15]. What is different is that in this study costs for services constituted nearly two-thirds of total costs, while in the other studies it was the reverse. Based on earlier qualitative fieldwork in the region, the VHW interviews, and an earlier study in the west of The Gambia [16], it could be hypothesized that the relatively high expenditure on services is due to regular stock-outs at health facilities that require people to purchase treatment at private pharmacies in the larger towns. This is a cause of frustration for VHWs who despite being trained to test and treat (uncomplicated) malaria, are often not able to do so because of a lack of RDTs and anti-malarial drugs. The majority of transport and services costs incurred by households for seeking malaria treatment could be avoided if people could consistently receive treatment locally in their own village. This would require more supplies and better distribution according to need.

Other drivers of treatment-seeking costs found in the literature are relative household wealth, public versus private facility, and availability of drugs at the health facility. In this study data on household wealth was not collected, but it is reasonable to postulate that involvement in business and educational level (which significantly affects total cost in this study’s sample) are proxies for household wealth. In addition, given the social structure in the Gambia, it is reasonable to assume that healthcare costs are shared by members of a household or a compound. This may mean that, although this study’s results did not show being a household or compound head has a significantly effect on costs, *actual* malaria-related costs for these people may be higher as they are expected to pay for the care of others under their care as well. The survey did not include questions specifically about if respondents went to a public or private health facility because there are almost no private clinics in URR and data on stock-outs at health facilities is not available. However, the relatively high costs of services in a system that should in theory provide malaria care for free could be a reflection of the fact that a part of treatment (drug provision) is being taken up by private pharmacies.

A major strength of this study is the large sample of respondents across URR and the inclusion of the VHW perspective. This provides confidence in the reliability of the results. The primary limitation of this study is the cross-sectional approach and the reliance on respondent call-back. Despite practical and financial barriers, the majority of respondents reported seeking biomedical treatment for malaria at a health facility. This is in line with earlier research in The Gambia [16, 17], but there are two limitations that put these findings into perspective [17]. First of all, there can be an overlap between (symptoms of) malaria and certain folk illnesses. Secondly, some symptoms of malaria can be perceived to have supernatural causes. The treatment-seeking behaviour for folk illnesses and supernatural afflictions are different than those for malaria, even though a malaria infection may be a cause for both of them. These factors may have led to an overestimation of health facility preference and an underestimation of the total malaria-related cost impact on URR. Relying on respondent recall may also have biased the costing results as respondents may not remember correctly what was spent a few months before the interview. There was no question that asked them about *when* the latest malaria episode occurred, which made it is hard to assess the impact of the recall bias on the results. A related limitation is that this survey was administered in between year 1 and year 2 of the MASSIV trial. Potential benefits derived from access to the study medicines could have added a desirability bias to participants’ responses. Another limitation is costs incurred for severe malaria. As this is relatively rare and is sometimes fatal, it is likely that the results of this study mainly reflect costs incurred for uncomplicated malaria. Finally, because this focused on out-of-pocket expenditures, the results primarily reflect financial rather than economics costs. Data was in fact collect data on working days lost, but converting this to a monetary value was not practical, as 80% of the respondents were subsistence farmers and thus had no formal income. There are methods for assessing wealth of subsistence farmers, but they are time-intensive and would have carried the risk of respondent fatigue in an already long survey.

## Conclusions

The residual transmission of malaria in The Gambia’s URR region requires households to keep spending money on prevention and treatment. This study has quantified the latter and found that costs are incurred for both transport and services, despite malaria care in The Gambia being provided free-of-charge and VHWs being available in most villages. To reduce malaria transmission in areas with a high coverage of prevention interventions, novel approaches such as mass drug administration with ivermectin are needed. This study can add to the evidence base that is needed to establish the cost-effectiveness of such approaches for regular use or for malaria elimination. Apart from clinical and costing data, cost-effectiveness studies and policy should take into account health system barriers and local cultural interpretations of disease that may prevent theoretically efficacious interventions from reaching their full potential for vulnerable populations.

## Data Availability

The dataset analysed for the current study is not publicly available due to confidentiality concerns; however, they are available from the corresponding author on reasonable request.
